# Blood lead level and risk of hypertension in the United States National Health and Nutrition Examination Survey 1999–2016

**DOI:** 10.1038/s41598-021-82435-6

**Published:** 2021-02-04

**Authors:** Man Fung Tsoi, Chris Wai Hang Lo, Tommy Tsang Cheung, Bernard Man Yung Cheung

**Affiliations:** 1 Department of Medicine, Queen Mary Hospital, The University of Hong Kong, 102 Pokfulam Road, Hong Kong, China; 2 Partner State Key Laboratory of Pharmaceutical Biotechnology, The University of Hong Kong, Hong Kong, China; 3 Institute of Cardiovascular Science and Medicine, The University of Hong Kong, Hong Kong, China; 4 Hong Kong Sanatorium & Hospital, Hong Kong, China

**Keywords:** Hypertension, Risk factors, Epidemiology

## Abstract

Lead is a heavy metal without a biological role. High level of lead exposure is known to be associated with hypertension, but the risk at low levels of exposure is uncertain. In this study, data from US NHANES 1999–2016 were analyzed. Adults with blood lead and blood pressure measurements, or self-reported hypertension diagnosis, were included. If not already diagnosed, hypertension was defined according to the AHA/ACC 2017 hypertension guideline. Results were analyzed using R statistics version 3.5.1 with sample weight adjustment. Logistic regression was used to study the association between blood lead level and hypertension. Odds ratio (OR) and 95% confidence interval (95% CI) were estimated. Altogether, 39,477 participants were included. Every doubling in blood lead level was associated with hypertension (OR [95%CI] 1.45 [1.40–1.50]), which remained significant after adjusting for demographics. Using quartile 1 as reference, higher blood lead levels were associated with increased adjusted odds of hypertension (Quartile 4 vs. Quartile 1: 1.22 [1.09–1.36]; Quartile 3 vs. Quartile 1: 1.15 [1.04–1.28]; Quartile 2 vs. Quartile 1: 1.14 [1.05–1.25]). In conclusion, blood lead level is associated with hypertension in the general population with blood lead levels below 5 µg/dL. Our findings suggest that reducing present levels of environmental lead exposure may bring cardiovascular benefits by reducing blood pressure.

## Introduction

Lead is a heavy metal widely used in industrial applications, but it does not have any biological role in humans. Due to its widespread use, humans are exposed to lead mainly through occupational exposure and drinking water^1^. Lead exposure is known to affect neurocognitive development in children^1^, and increase the risk of cardiovascular disease (CVD) in adults^2^. Although not ideal, blood lead level is measured as an indicator of exposure and toxicity. It is now acknowledged that there is no safe level of exposure as far as lead is concerned. The current upper reference level in the United States (US) is 5 µg/dL according to the Centers for Disease Control and Prevention (CDC)^3^. Using data from the US National Health Nutrition and Examination Survey (NHANES), we previously demonstrated a continual decline in blood lead level in 1999–2014^4^. The 97.5 percentile of blood lead level in children aged 1–5 was 3.48 µg/dL^4^, which suggested that the upper reference level of blood lead should be revised to reflect the declining blood lead level in US children. Because lead is toxic even at low blood levels^1^, reducing lead in the environment and human exposure remains an important issue to be addressed^5^.

In adults, lead is also harmful. It increases reactive oxygen species production, activates nuclear factor-κB and causes inflammation, resulting in endothelial injury and vascular dysfunction^6^. Changes in the autonomic nervous system may also play a role in CVD development^1,6^. Several epidemiological studies have shown a positive association between blood lead level and cardiovascular mortality, stroke and myocardial infarction^7–11^.

In particular, lead increases the risk of hypertension. High blood lead level is associated with an increased risk of hypertension in epidemiological studies^12^ and meta-analyses^2,13^. However, the association at low blood lead levels is uncertain^14–18^.

A significant relationship between blood lead level and odds for hypertension was reported in studies of the Brazilian (OR 2.54, 95% CI 1.17–5.53)^14^ and American population (OR 2.69, 95% CI 1.08–6.72)^15^. This relationship has also been reported in a Korean study^16^. On the contrary, another American study showed conflicting results using odds of hypertension as an outcome (OR 0.95, 95% CI 0.90–1.01)^17^. A prospective Swedish study showed elevated hypertension incidence at baseline (OR 1.3, 95% CI 1.1–1.5), but the association diminished upon prospective follow up^18^. Therefore, we conducted this study to confirm the association between hypertension and blood lead level in the range typically found in the general population, using data from the latest NHANES.

## Methods

In this study, we used the US NHANES sample population as representative of the US population^19^. Each participant represents approximately 50,000 individuals. The NHANES study was approved by the National Center for Health Statistics Research Ethics Review Board of the CDC in the US. All participants gave informed consent.

We included adult participants aged ≥ 20 years with blood lead and blood pressure measurements in NHANES 1999–2016. 10,065 participants without blood lead or blood pressure measurements were excluded. Those who did not respond to the question “Have you ever been told by a doctor or other health professional that you had hypertension, also called high blood pressure?” were also excluded.

Venous blood was obtained to measure blood lead level. Blood samples were stored at − 20 °C before measurement. The measurement of blood lead level was conducted using Inductively Coupled Plasma Dynamic Reaction Cell Mass Spectrometer (ELAN DRC II, PerkinElmer, Norwalk) in the central laboratory according to the standard protocol^19^. The lower limits of detection were 0.3 µg/dL in 1999–2002, 0.28 µg/dL in 2003–2004, 0.25 µg/dL in 2003–2012, and 0.07 µg/dL in 2013–2016. All blood lead levels lower than the lower limit of detection were replaced by the lower limit of detection divided by √2^20^.

Blood pressure was measured according to a standard protocol^19^. Participants were required to have a rest period of 5 min before the first blood pressure reading. The Baumanometer calibrated mercury true gravity sphygmomanometer with Baumanometer Calibrated V-Lok cuffs with Latex Inflation Bulb, Air-Flo Control Valve was used to measure the blood pressure of participants. Blood pressure readings were extracted according to specifications from the analytical note of US NHANES. If only one blood pressure reading was obtained, the readings was the blood pressure recorded. If there was more than one pressure reading, the first one was always excluded. The blood pressure recorded was the average of the readings after excluding the first reading.

Hypertension was defined as self-reported hypertension or self-reported anti-hypertensive medication prescription in the question “Are you now taking prescribed medicine.” If participants did not have self-reported hypertension, their blood pressure readings in NHANES were used to define hypertension according to the American Heart Association/American College of Cardiology (AHA/ACC) 2017 guideline for monitoring, diagnosis of hypertension^21^. Participants with systolic blood pressure of ≥ 130 mmHg or diastolic blood pressure ≥ 80 mmHg were considered hypertensive^21^.

Results were analyzed using R statistics version 3.5.2^22^ and the package “survey” version 3.34^23^. Sample weights were used to adjust for unequal probability of distribution, oversampling and sampling bias. The demographic characteristics across different survey cycles were estimated using sample weights. Chi-square tests and multiple regression were used to analyze the categorical and continuous variables, respectively, expressed in p-value with a significance level of 0.001. Multiple regression was used to estimate the effect of doubling in blood lead level on systolic blood pressure. Regression coefficients and their 95% confidence intervals (95% CI) were estimated. Logistic regression was used to estimate the association between every doubling in blood lead level and the prevalence of hypertension. Odds ratio and 95% CI were estimated. Further analysis was conducted on the association between blood lead level and hypertension according to the quartiles of blood lead level, gender and ethnicity.

Demographic variables were included in regression models as covariates, including age, gender (male or female), ethnicity (Mexican American, Other Hispanics, Non-Hispanic White, Non-Hispanic Black, and other ethnicities), waist circumference (in centimeters) and ever cigarette smoking. Odds ratios and regression coefficients were adjusted for the appropriate covariates as described in Tables 3 and 4 and Supplementary Table 2–4.

### Ethical approval

All methods used in the manuscript were carried out in accordance with guidelines and regulations cited in this study.

## Results

Altogether, 39,447 participants were included in this analysis. Their characteristics are summarized in Table 1 and 2. Compared to participants without hypertension, hypertensive participants had a higher proportion of males, higher blood lead level and waist circumference. The characteristics of participants according to quartiles of blood lead level are summarized in Supplementary Table 1. The prevalence of hypertension increased with quartiles of blood lead level (p < 0.001). The blood lead levels ranged from 0.005 to 23.51 µg/dL amongst participants.

**Table 1:** Characteristics of participants included in this study.

	1999–2000	2001–2002	2003–2004	2005–2006	2007–2008	2009–2010	2011–2012	2013–2014	2015–2016	p
**N**	4207	4772	4525	4509	5364	5765	5030	2695	2610	
**Age**^**a**^	45.01 ± 0.38	45.46 ± 0.52	46.40 ± 0.52	46.78 ± 0.73	47.05 ± 0.43	47.07 ± 0.49	47.43 ± 0.81	47.45 ± 0.42	47.98 ± 0.80	< 0.001
**Male (%)**^**b**^	47.9 (46.3–49.0)	47.8 (46.7–49.0)	48.2 (46.7–50.0)	47.9 (46.5–49.0)	48.0 (46.8–49.0)	48.2 (47.0–49.0)	47.9 (46.2–50.0)	48.1 (46.6–50.0)	48.1 (45.2–51.0)	0.999
**Ethnicity (%)**^**b**^										
Mexican Americans	6.32 (3.97–10.00)	7.17 (5.46–9.00)	7.70 (4.46–13.00)	7.96 (6.10–10.00)	8.41 (5.72–12.00)	8.54 (4.94–14.00)	7.76 (4.77–12.00)	9.15 (5.74–14.00)	8.72 (5.02–15.00)	0.666
Other Hispanics	8.28 (3.84–17.0)	5.87 (2.92–11.00)	3.62 (2.50–5.00)	3.30 (2.01–5.00)	4.93 (2.89–8.00)	5.03 (2.96–8.00)	6.52 (3.96–11.00)	5.54 (3.62–8.00)	6.55 (4.18–10.00)	
Non-Hispanic White	70.60 (64.50–76.00)	72.20 (66.30–77.00)	72.50 (64.80–79.00)	72.50 (66.30–78.00)	70.10 (61.80–77.00)	68.60 (61.10–75.00)	67.10 (58.50–75.00)	65.80 (58.00–73.00)	64.20 (55.80–72.00)	
Non-Hispanic Black	10.47 (7.46–14.00)	10.35 (7.26–15.00)	11.10 (7.59–16.00)	10.90 (7.66–15.00)	10.40 (7.10–15.00)	10.87 (9.25–13.00)	11.01 (7.12–17.00)	11.20 (7.90–16.00)	11.41 (7.66–17.00)	
Other ethnicities	4.31 (2.53–7.00)	4.46 (3.20–6.00)	5.16 (4.06–7.00)	6.20 (4.26–9.00)	6.93 (4.94–10.00)	7.62 (5.60–10.00)	5.30 (4.04–7.00)	8.23 (6.66–10.00)	9.15 (6.82–12.00)	
**Blood lead Level (µg/dL)**^**c**^	1.75 [1.69–1.81]	1.56 [1.50–1.62]	1.52 [1.45–1.59]	1.41 [1.34–1.48]	1.38 [1.31–1.45]	1.23 [1.19–1.27]	1.09 [1.03–1.16]	0.97 [0.92–1.01]	0.92 [0.87–0.98]	< 0.001
**Waist circumference (cm)**^**a**^	95.43 ± 0.43	95.91 ± 0.33	97.37 ± 0.37	97.70 ± 0.64	97.91 ± 0.44	98.26 ± 0.43	98.79 ± 0.58	99.23 ± 0.54	100.7 ± 0.71	< 0.001
**Ever cigarette smoking (%)**^**b**^	47.9 (46.3–49.0)	47.8 (46.7–49.0)	48.2 (46.7–50.0)	47.9 (46.5–49.0)	48.0 (46.8–49.0)	48.2 (47.0–49.0)	47.9 (46.2–50.0)	48.1 (46.6–50.0)	48.1 (45.2–51.0)	0.001

^a^Figures are expressed as mean ± standard error (for mean age and waist circumference).

^b^Figures are expressed as percent (95% confidence intervals) (for gender, ethnicity and ever cigarette smoking).

^c^Figures are expressed as mean blood lead levels (95% confidence intervals).

**Table 2:** Characteristics of participants included in this study (subgroup analysis by hypertension).

	With hypertension	Without hypertension	p
**N**	20,803	18,674	
**Age**^**a**^	54.08 ± 0.23	39.87 ± 0.19	< 0.001
**Male (%)**^**b**^	51.6 (50.7–52.0)	44.7 (43.8–45.5)	< 0.001
**Ethnicity (%)**^**b**^			
Mexican Americans	6.1 (5.1–7.0)	9.9 (8.6–11.0)	< 0.001
Other Hispanics	4.4 (3.6–5.0)	6.6 (5.6–8.0)	
Non-Hispanic White	71.1 (68.7–73.0)	67.2 (64.8–69.0)	
Non-Hispanic Black	12.5 (11.1–14.0)	9.3 (8.3–10.0)	
Other ethnicities	6.0 (5.3–7.0)	7.0 (6.3–8.0)	
**Blood lead level (µg/dL)**^**c**^	1.44 [1.41–1.47]	1.12 [1.10–1.14]	< 0.001
**Waist circumference (cm)**^**a**^	102.94 ± 0.20	93.41 ± 0.21	< 0.001
**Ever cigarette smoking (%)**^**b**^	49.4 (48.1–51.0)	43.8 (42.5–45.0)	< 0.001
**Systolic blood pressure (mmHg)**^**a**^	129.7 ± 0.29	106.1 ± 0.36	< 0.001
**Diastolic blood pressure (mmHg)**^**a**^	73.1 ± 0.25	63.4 ± 0.19	< 0.001

^a^Figures are expressed as mean ± standard error (for mean age, waist circumference, systolic blood pressure and diastolic blood pressure).

^b^Figures are expressed as percent (95% confidence intervals) (for gender, ethnicity and ever cigarette smoking).

^c^Figures are expressed as mean blood lead levels (95% confidence intervals).

The regression coefficient for the effect of every doubling of blood lead level on systolic blood pressure is summarized in Supplementary Table 2. Every doubling of blood lead level was associated with a 3.25 [95% CI 2.94–3.55] mmHg increase in systolic blood pressure. This trend is also significant after adjustment for age, gender, ethnicity, waist circumference, poverty to income ratio, education, ever cigarette smoking, diabetes and stage 3–5 chronic kidney diseases (regression coefficient [95% CI] 0.52 [0.19–0.86]; Supplementary Table 2).

The association between every doubling in blood lead level and hypertension is summarized in Table 3. Every doubling of blood lead level was associated with an increase in hypertension risk (OR [95% CI] 1.45 [1.40–1.50]). This trend remained significant after adjustment as described above (1.09 [1.04–1.14]; Table 3).

**Table 3:** Association between blood lead level and hypertension.

	Odds ratio for every doubling in blood lead level [95% Confidence Interval]
Crude	1.45 [1.40–1.50]
Model 1	0.98 [0.95–1.02]
Model 2	1.10 [1.06–1.14]
Model 3	1.07 [1.02–1.13]
Model 4	1.09 [1.04–1.14]

Figures are expressed as odds ratio [95% confidence interval].

Model 1: adjusted for age, gender and ethnicity.

Model 2: adjusted for age, gender, ethnicity and waist circumference.

Model 3: adjusted for age, gender, ethnicity, waist circumference, poverty to income ratio, education and ever cigarette smoking.

Model 4: adjusted for age, gender, ethnicity, waist circumference, poverty to income ratio, education, ever cigarette smoking, diabetes and stage 3–5 chronic kidney diseases.

The association between quartiles of blood lead level and hypertension is summarized in Table 4. Using Quartile 1 of blood lead level, < 0.89 µg/dL as reference, blood lead level ≥ 0.89 µg/dL was associated with increased hypertension risk (OR [95% CI] 1.62 [1.49–1.75], 2.05 [1.89–2.23] and 2.59 [2.37–2.83] for Quartile 2, Quartile 3 and Quartile 4, respectively; Table 4). This trend remained significant after adjustment as described above (Table 4). The only exception was found for Quartile 3, in which the association did not reach statistical significance (1.13 [0.99–1.29]) after adjusting for age, gender, ethnicity, waist circumference, poverty to income ratio, education and ever cigarette smoking.

**Table 4:** Association between quartiles of blood lead level and hypertension.

	Quartile 1	Quartile 2	Quartile 3	Quartile 4
Blood lead level (µg/dL)	< 0.89	0.89–< 1.30	1.30–< 2.10	≥ 2.10
Crude	1 (reference)	1.62 [1.49–1.75]	2.05 [1.89–2.23]	2.59 [2.37–2.83]
Model 1	1 (reference)	1.01 [0.92–1.10]	0.96 [0.87–1.05]	0.95 [0.87–1.04]
Model 2	1 (reference)	1.15 [1.05–1.25]	1.17 [1.06–1.29]	1.25 [1.13–1.38]
Model 3	1 (reference)	1.13 [1.01–1.26]	1.13 [0.99–1.29]	1.16 [1.01–1.39]
Model 4	1 (reference)	1.15 [1.04–1.26]	1.17 [1.05–1.31]	1.21 [1.07–1.36]

Figures are expressed as odds ratio [95% confidence interval].

Model 1: adjusted for age, gender and ethnicity.

Model 2: adjusted for age, gender, ethnicity and waist circumference.

Model 3: adjusted for age, gender, ethnicity, waist circumference, poverty to income ratio, education and ever cigarette smoking.

Model 4: adjusted for age, gender, ethnicity, waist circumference, poverty to income ratio, education, ever cigarette smoking, diabetes and stage 3–5 chronic kidney diseases.

The association between every doubling of blood lead level and hypertension stratified by gender is summarized in Supplementary Table 3. Every doubling of blood lead level was associated with an increased risk in hypertension in both men (OR [95% CI] 1.25 [1.20–1.30]) and women (1.65 [1.56–1.74]). The association was statistically significant in men after adjusting for demographic variables mentioned above except gender itself (1.10 [1.05–1.16]). The associations between blood lead and hypertension failed to reach statistical significance in women when further adjusted for demographic variables (Model 4: 1.04 [0.97–1.11]).

The association between every doubling of blood lead level and hypertension stratified by ethnicity is summarized in Supplementary Table 4. Every doubling of blood level was associated with an increased risk of hypertension for all ethnicities before adjustment, but did not reach statistical significance after adjusting for demographic variables. The only exception was in the non-Hispanic white subgroups, in which the association remained statistically significant after adjusting for age, gender, waist circumference, poverty-to-income ratio, education, ever cigarette smoking, diabetes and stage 3–5 chronic kidney diseases (OR [95% CI] 1.12 [1.05–1.19]).

## Discussion

Our study demonstrated a significant association between blood lead level and hypertension. The association remained significant after adjustment. In US NHANES, the sample size was large, and both the blood lead and blood pressure levels were representative of the general population. Measurements were performed according to a strict protocol with stringent quality control. Moreover, the inclusion of participants drawn from the general population meant that the vast majority of these participants had blood levels below the upper reference range. Therefore, our finding of an association between blood pressure and blood lead level applies to the whole population rather than people with elevated blood lead level only.

A doubling in blood lead level, even in the normal range, is associated with an increase in blood pressure. Although the associated increase is very small, we need to bear in mind that in clinical trials and meta-analysis, an increase of 2 mmHg in the systolic blood pressure already increases the stroke risk^24^. Moreover, elevations in the blood lead level occur not so much in isolated unlucky individuals but in whole households and districts. The most famous example was in Flint, Michigan^25^. The cumulative impact of elevated blood level at a population level can therefore be enormous. Therefore, despite a continual decline in blood lead level^5^, the harmful effects of lead should not be overlooked from a public health perspective.

In sex-stratified analysis, we found that the association between blood lead and hypertension remained significant in the fully-adjusted model in men but not in women. Gender-related differences in blood pressure are well known^26^, with the latest epidemiological data again showing a higher prevalence of hypertension in men^27^. Industrial occupation is a major source of lead exposure^28^. Men are more likely to be engaged in manual labor jobs such as mining and construction.

In ethnicity-stratified analysis, we did not find statistically significant associations between blood lead and hypertension in all ethnic subgroups, except for non-Hispanic whites. These associations did not reach statistical significance because of reduced statistical power. Ethnic differences in the relationship between blood lead and hypertension have been found in other studies^15,17^.

Our study adds to the accumulating evidence that lead exposure is related to high blood pressure, as demonstrated in populations in Brazil^14^, US^15,29^ and Korea^16^. Our findings are consonant with a recent analysis of NHANES 1999–2016 data that showed an association of blood lead level with uncontrolled hypertension^30^. A Swedish study also found an association between blood lead and hypertension, although the association diminished over time during the follow-up period^18^. In contrast, a previous study using NHANES 2003–2010 data did not demonstrate a significant association between blood lead level and hypertension^17^. Our study had a larger sample size, and we used the new US definition of hypertension, which increases the number of people deemed to be hypertensive.

Blood lead has a half-life of about a month^31^, whereas bone lead has a half-life of over 10 years^32^. Lead is slowly released from cortical bone via osteoclast resorption^32^, and can induce insidious oxidative stress and damage. Bone serves as the major reservoir for lead in the body^32,33^, and so bone lead level is the more ideal marker of chronic exposure^32^. However, it is determined indirectly using X-ray fluorescence^32^ and was not measured in NHANES.

Besides occupational exposure^28^, the population is exposed to lead in different ways. Leaded gasoline was once a major source for lead exposure^34^. Thus, households near major roads and in cities are more exposed to lead from gasoline. However, leaded gasoline has been phased out in recent years^5^. Other sources of lead exposure include lead paint, lead pipes and industrial exposure^5,34^. Lead paints and lead water pipes are no longer used, but they could still be present in older houses.

The mechanisms by which lead increases blood pressure have not been fully elucidated. Besides oxidative stress, lead has been postulated to reduce bioactive nitrogen monoxide (NO) and downregulate soluble guanylate cyclase in vascular tissues^6^. It causes a reduction in cyclic GMP production and attenuation of NO activity, leading to vascular remodeling and inhibition in vasorelaxation^6^. Lead also competes with calcium for transport in the human body via ion channels^6,35^. This contributes to changes in cytosolic calcium ion level, which regulate vascular tone^35^. These molecular effects combine for increased vascular resistance. Lead is proposed to increase angiotensin-converting enzyme activity as well^36^, and thus increase in blood pressure.

Despite its strength, this study is not without limitations. While we can demonstrate a cross-sectional association, we cannot draw any conclusions on the long-term effects of lead on the development of hypertension. The data linking blood lead levels with hypertension, including this study, comes from observational data. Therefore, causation cannot be inferred from these data alone. Furthermore, the US has a long history of industrialization and use of leaded gasoline in automobiles. The risks posed by lead may be different in other countries. Hypertension is caused by multiple factors, including genetic and environmental factors, and lead is only one of many such environmental factors.

## Conclusions

Blood lead level is associated with hypertension in the general population, most of whom did not have elevated blood levels. Our findings suggest that reducing present levels of environmental lead exposure may benefit adults by reducing blood pressure and its attendant cardiovascular risk.

## Supplementary Information

Supplementary Tables.
