# Supplementary material for: Blood lead level and risk of hypertension in the United States National Health and Nutrition Examination Survey 1999–2016
Source: Sci Rep. 2021 Feb 4;11:3010. doi: 10.1038/s41598-021-82435-6 (PMC7862639; doi:10.1038/s41598-021-82435-6)
Supplement: Supplementary file 1 — Supplementary Tables. [file 41598_2021_82435_MOESM1_ESM.docx]

**Blood Lead Level and Risk of Hypertension in the United States National Health and Nutrition Examination Survey 1999-2016**

Man Fung Tsoi, PhD;^1,5^ Chris Wai Hang Lo;^1,5^ Tommy Tsang Cheung, FRCPE;^1,3,4,5^ Bernard Man Yung Cheung, FRCP^1,2,3,5,*^

^1^Department of Medicine

^2^Partner State Key Laboratory of Pharmaceutical Biotechnology

^3^Institute of Cardiovascular Science and Medicine

The University of Hong Kong

^4^Hong Kong Sanatorium & Hospital

^5^This author takes responsibility for all aspects of the reliability and freedom from bias of the data presented and their discussed interpretation.

*This author is the corresponding author.

**Correspondence**

Prof. Bernard Cheung

University Department of Medicine

Queen Mary Hospital

The University of Hong Kong

102 Pokfulam Road

Email: mycheung@hku.hk

Tel: +852 22554347

Fax: +852 28186474

**Keywords:** Blood lead level, Hypertension, NHANES

**Supplementary Files**

Supplementary Table 1. Characteristics of participants included in this analysis according to quartiles of blood lead level

|  | Quartile 1 | Quartile 2 | Quartile 3 | Quartile 4 | p |
| --- | --- | --- | --- | --- | --- |
| N | 9736 | 9468 | 10388 | 9885 |  |
| Blood lead Level (µg/dL) | <0.89 | 0.89-<1.30 | 1.30-<2.10 | ≥2.10 |  |
| Age^a^ | 38.33±0.28 | 46.38±0.29 | 51.20±0.26 | 54.80±0.30 | <0.001 |
| Male (%)^b^ | 33.1 (32.0-34.0) | 45.4 (44.1-47.0) | 54.5 (53.2-56.0) | 66.0 (64.7-67.0) | <0.001 |
| Ethnicity (%)^b^ |  |  |  |  |  |
| Mexican Americans | 8.9 (7.5-11.0) | 7.2 (6.1-8.0) | 7.5 (6.4-9.0) | 8.4 (7.1-10.0) | <0.001 |
| Other Hispanics | 6.6 (5.6-8.0) | 5.6 (4.6-7.0) | 4.9 (3.9-6.0) | 4.5 (3.3-6.0) |  |
| Non-Hispanic White | 67.9 (65.3-70.0) | 70.0 (67.5-72.0) | 70.4 (68.0-73.0) | 68.0 (65.1-71.0) |  |
| Non-Hispanic Black | 10.8 (9.3-12.0) | 10.2 (9.1-11.0) | 10.5 (9.3-12.0 | 12.5 (11.0-14.0) |  |
| Other ethnicities | 5.8 (5.1-7.0) | 7.0 (6.2-8.0) | 6.7 (5.9-8.0) | 6.7 (5.7-8.0) |  |
| Waist circumference (cm)^a^ | 98.22±0.32 | 98.03±0.26 | 98.08±0.25 | 97.65±0.23 | 0.714 |
| Ever cigarette smoking (%)^b^ | 31.7 (30.0-33.0) | 44.1 (42.4-46.0) | 53.7 (52.0-55.0) | 63.7 (62.2-65.0) | <0.001 |
| Hypertension (%)^b^ | 36.9 (35.3-38.0) | 48.5 (47.0-50.0) | 54.5 (53.1-56.0) | 60.2 (58.5-62.0) | <0.001 |

Supplementary Table 1

^a^ Figures are expressed as mean ± standard error (for mean age and waist circumference).

^b^ Figures are expressed as percent (95% confidence intervals) (for gender, ethnicity, ever cigarette smoking and hypertension).

Supplementary Table 2. Effect of doubling blood lead level on systolic blood pressure

|  | Regression Coefficient [95% Confidence Interval] |
| --- | --- |
| Crude | 3.25 [2.94-3.55] |
| Model 1 | 0.36 [0.05-0.66] |
| Model 2 | 0.73 [0.42-1.03] |
| Model 3 | 0.58 [0.24-0.93] |
| Model 4 | 0.52 [0.19-0.86] |

Supplementary Table 2

Figures are expressed as odds ratio [95% confidence interval].

Statistical adjustment for Supplementary Table 2

Model 1: adjusted for age, gender and ethnicity

Model 2: adjusted for age, gender, ethnicity and waist circumference

Model 3: adjusted for age, gender, ethnicity, waist circumference, poverty to income ratio, education and ever cigarette smoking

Model 4: adjusted for age, gender, ethnicity, waist circumference, poverty to income ratio, education, ever cigarette smoking, diabetes and stage 3-5 chronic kidney diseases

Supplementary Table 3. Gender-specific analysis for the association between every doubling of blood lead level and hypertension

|  | Male | Female |
| --- | --- | --- |
| Crude | 1.25 [1.20-1.30] | 1.65 [1.56-1.74] |
| Model 1 | 1.00 [0.96-1.04] | 0.94 [0.88-1.00] |
| Model 2 | 1.08 [1.03-1.14] | 1.03 [0.97-1.10] |
| Model 3 | 1.08 [1.03-1.14] | 1.03 [0.96-1.10] |
| Model 4 | 1.10 [1.05-1.16] | 1.04 [0.97-1.11] |

Supplementary Table 3

Figures are expressed as odds ratio [95% confidence interval].

Statistical adjustment for Supplementary Table 3

Model 1: adjusted for age and ethnicity

Model 2: adjusted for age, ethnicity and waist circumference

Model 3: adjusted for age, ethnicity, waist circumference, poverty to income ratio, education and ever cigarette smoking

Model 4: adjusted for age, ethnicity, waist circumference, poverty to income ratio, education, ever cigarette smoking, diabetes and stage 3-5 chronic kidney diseases

Supplementary Table 4. Ethnicity-specific analysis for the association between every doubling of blood lead level and hypertension

|  | Mexican Americans | Other Hispanics | Non-Hispanic Whites | Non-Hispanic Blacks | Other ethnicities |
| --- | --- | --- | --- | --- | --- |
| Crude | 1.15 [1.08-1.21] | 1.34 [1.21-1.49] | 1.51 [1.44-1.57] | 1.56 [1.47-1.65] | 1.31 [1.17-1.46] |
| Model 1 | 0.90 [0.84-0.97] | 0.94 [0.83-1.08] | 1.00 [0.96-1.05] | 0.99 [0.93-1.05] | 0.94 [0.83-1.08] |
| Model 2 | 0.98 [0.89-1.07] | 1.03 [0.90-1.19] | 1.09 [1.04-1.15] | 1.04 [0.97-1.12] | 1.09 [0.95-1.26] |
| Model 3 | 0.97 [0.88-1.06] | 1.06 [0.92-1.22] | 1.10 [1.04-1.17] | 1.05 [0.97-1.13] | 1.10 [0.95-1.28] |
| Model 4 | 0.98 [0.89-1.08] | 1.07 [0.93-1.23] | 1.12 [1.05-1.19] | 1.06 [0.99-1.15] | 1.10 [0.95-1.28] |

Supplementary Table 4

Figures are expressed as odds ratio [95% confidence interval].

Statistical adjustment for Supplementary Table 4

Model 1: adjusted for age and gender

Model 2: adjusted for age, gender and waist circumference

Model 3: adjusted for age, gender, waist circumference, poverty to income ratio, education and ever cigarette smoking

Model 4: adjusted for age, gender, waist circumference, poverty to income ratio, education, ever cigarette smoking, diabetes and stage 3-5 chronic kidney diseases
